# Computational and structural biotechnology meets nanoscience and advanced materials

**DOI:** 10.1016/j.csbj.2023.12.012

**Published:** 2023-12-14

**Authors:** Antreas Afantitis, Iseult Lynch

**Affiliations:** Nanoinformatics Department, NovaMechanics Ltd, Nicosia, Cyprus; Entelos Institute, Larnaca, Cyprus; Entelos Institute, Larnaca, Cyprus; School of Geography, Earth and Environment Sciences, University of Birmingham, Edgbaston, B15 2TT Birmingham, United Kingdom; Centre for Environmental Research and Justice (CERJ), University of Birmingham, Edgbaston, B15 2TT Birmingham, United Kingdom

The launch of a new section of Computational and Structural Biotechnology Journal covering the breath of Nanoscience and Advanced Materials (NAM) provides a unique platform for reporting experimental and computational studies on the exciting science that occurs at the interface between engineered and manufactured materials and living systems across scales. This innovative platform is dedicated to bridging the gaps and fostering intersections between various scientific disciplines such as nanoscience, materials science, chemistry, physics, and biomedical engineering. Our goal is to catalyze scientific knowledge and technological innovation, driving transformative advances in the field of nanomaterials and advanced materials. The fascinating science at the bio-nano interface [1], [2], which includes exploration of the protein corona [3], [4], biomolecule corona [5] or environmental corona [6] - the layer of biomolecules that overlays a biological identity onto the synthetic identity provided by the chemical composition and morphology of the materials or surfaces, has been the subject of intensive experimental and computational research over the last decade or more [7], [8]. The role of the bio-nano interface in medicine, agriculture, environmental remediation, sensing, catalysis, and energy capture is increasingly recognized, yet full understanding of the impacts of biomolecule-material interactions for both the biomolecules involved and for the nanoscale or advanced materials remains elusive. Experimental and computational exploration of the bio-nano interface offers enormous opportunities for new insights and new understanding, in a range of important processes such as biofouling, acceptance or rejection of biomaterials and implants, biosensing and more. The role of the biomolecule corona in receptor binding is increasingly understood [9], providing the key to understanding transport across biological barriers and engagement of biological signalling pathways, including those leading to adverse (health) outcomes [10]. The ability to generate fully computational nanomaterials, for example as digital twins, offers exciting new avenues for in silico assessment of bio-nano interactions and the molecular pathways activated in response to nanotherapeutics, and those activated by nanomaterials identified as foreign bodies resulting from pollution. The NAM section of CSBJ welcomes these advances and more.

Beyond bio-nano interactions, the NAM section of CSBJ focuses on advancing the scientific knowledge and technological innovation at the crossroads of nanoscience, materials science, chemistry, physics, and biomedical engineering. The NAM section is more than just a repository for research; it's a hub for pioneering ideas and groundbreaking discoveries. We welcome contributions from diverse spheres, ranging from academia, to regulatory and policy researchers, and industry professionals, each bringing unique insights to our collective understanding of the nanoscale world and its applications. Our section will cover a broad spectrum of topics within nanoscience and advanced materials. These include, but are not limited to:

1. Nanotechnology Applications:

From Nanocatalysis to Nanofabrication, and Nanophotonics to Biomedical Nanotechnology, we delve into the myriad applications of nanoscience in various fields, including agriculture and environmental technology.

2. Risk Management and Governance:

Emphasizing sustainable, safe design strategies and smart materials, this area explores the intersection of bio-inspired materials and green chemistry, focusing on responsible innovation and governance in nanotechnology.

3. Data and Modeling:

With a focus on predictive toxicogenomics, nano(chem)informatics, and multiscale simulations, this domain aims to harness the power of AI, machine learning, and digital platforms to revolutionize our understanding and application of nanomaterials.

4. Risk Assessment and Integrated Approaches:

A critical aspect of our section involves comprehensive assessments of hazards, exposure, and risk management in the lifecycle of nanomaterials, underpinned by advanced data management and modeling techniques.

The Editor in Chief and Editorial board are looking forward to welcoming submissions covering the full spectrum of topics within the overarching domains of nanoscience and advanced materials, enhancing our understanding of the nanoscale world and its transformative applications. Potential contributors are also welcome to suggest topics for thematic issues and to guest-edit these. For example, the NAMs section of CSBJ is currently accepting submissions for a special issue on “*Applications of Cheminformatics and Machine Learning in Predictive Modelling of Property and Toxicity Endpoints of Nanoparticles*”, which is being guest edited by Prof. Kunal Roy from Jadavpur University, India. If you are working on this topic, please submit your articles by 31st August 2024. We are especially interested in well-executed and well-written no-effects studies, as reporting of the absence of nanomaterials or advanced materials toxicity is critical to the development of balanced predictive models and to developing a representative regulatory framework. Watch out for other exciting topical collections over the coming months. As we embark on this journey, your contributions, insights, and innovations will be the driving force behind the success of CSBJ: Nanoscience and Advanced Materials. We eagerly await your submissions and are excited to see how your work will contribute to this ever-evolving field.
